# Activation of the Peroxisome Proliferator-Activated Receptors (PPAR-*α*/*γ*) and the Fatty Acid Metabolizing Enzyme Protein CPT1A by Camel Milk Treatment Counteracts the High-Fat Diet-Induced Nonalcoholic Fatty Liver Disease

**DOI:** 10.1155/2021/5558731

**Published:** 2021-07-09

**Authors:** Haifa M. AlNafea, Aida A. Korish

**Affiliations:** ^1^Clinical Laboratory Sciences Department, College of Applied Medical Sciences, King Saud University, Unit No. 3928, PO Box 7960, Riyadh 12284, Saudi Arabia; ^2^Physiology Department (29), College of Medicine, King Saud University Medical City (KSUMC), King Saud University, PO Box 2925, Riyadh 11461, Saudi Arabia

## Abstract

Camel milk (CM) has a unique composition rich in antioxidants, trace elements, immunoglobulins, insulin, and insulin-like proteins. Treatment by CM demonstrated protective effects against nonalcoholic fatty liver disease (NAFLD) induced by a high-fat cholesterol-rich diet (HFD-C) in rats. CM dampened the steatosis, inflammation, and ballooning degeneration of the hepatocytes. It also counteracted hyperlipidemia, insulin resistance (IR), glucose intolerance, and oxidative stress. The commencement of NAFLD triggered the peroxisome proliferator-activated receptor-*α* (PPAR-*α*), carnitine palmitoyl-transferase-1 (CPT1A), and fatty acid-binding protein-1 (FABP1) and decreased the PPAR-*γ* expression in the tissues of the animals on HFD-C. This was associated with increased levels of the inflammatory cytokines IL-6 and TNF-*α* and leptin and declined levels of the anti-inflammatory adiponectin. Camel milk treatment to the NAFLD animals remarkably upregulated PPARs (*α*, *γ*) and the downstream enzyme CPT1A in the metabolically active tissues involved in cellular uptake and beta-oxidation of fatty acids. The enhanced lipid metabolism in the CM-treated animals was linked with decreased expression of FABP1 and suppression of IL-6, TNF-*α*, and leptin release with augmented adiponectin production. The protective effects of CM against the histological and biochemical features of NAFLD are at least in part related to the activation of the hepatic and extrahepatic PPARs (*α*, *γ*) with consequent activation of the downstream enzymes involved in fat metabolism. Camel milk treatment carries a promising therapeutic potential to NAFLD through stimulating PPARs actions on fat metabolism and glucose homeostasis. This can protect against hepatic steatosis, IR, and diabetes mellitus in high-risk obese patients.

## 1. Introduction

The global upsurge in the incidence of obesity, type II diabetes mellitus (DM), and the metabolic syndrome has boosted the incidence of nonalcoholic fatty liver disease (NAFLD) [1]. Fatty liver affects about 25% of the population worldwide, and the magnitude of the problem is larger in the Middle East due to the higher prevalence of obesity [2]. Multiple risk factors have been linked with the incidence of NAFLD including: genetic predisposition, lack of physical activity, high caloric intake, oxidative stress, inflammatory cytokines, gut infections, and impaired immune response [3, 4].

The first stage of the pathophysiology of NAFLD involves increased fat deposition in the hepatocytes which is referred to as hepatic steatosis. This can progress to nonalcoholic steatohepatitis (NASH) characterized by more susceptibility to hepatocyte injury and death by inflammation, oxidative stress, gut bacterial endotoxins, and mitochondrial dysfunction [5]. Consequently, activation of the hepatic stellate cells increases extracellular matrix deposition leading to fibrosis and predisposes to cirrhosis, liver transplantation, and hepatic carcinoma [6].

Body fat metabolism and energy balance are regulated by a family of ligand-activated nuclear receptors called peroxisome proliferator-activated receptors (PPARs) that involve alpha (*α*), beta (*β*), gamma (*γ*), and delta (*δ*) subtypes [7]. PPARs are expressed in different metabolically active tissues, including the liver, heart, and the kidneys, in addition to skeletal muscles and brown fats [8]. Activation of PPARs has been involved in controlling the cellular uptake and metabolism of free fatty acids (FFAs), phospholipids, and cholesterol. The augmented lipid metabolism in response to PPAR activation takes place through repressing or suppressing multiple genes responsible for beta-oxidation, lipogenesis, lipolysis, and lipid transformation [9].

Each of the subtypes of PPARs could bind to and become variably stimulated by multiple endogenous molecules including complex lipids, fatty acids, and eicosanoids. Additionally, some environmental factors and pharmacological agents could also activate these receptors [10]. After attachment to their ligands, PPARs form a complex with retinoid X receptors (RXR) and binds to the nuclear peroxisome proliferator response element (PPRE) to regulate the gene expression of the enzyme proteins involved in insulin sensitivity, fatty acid (FA) uptake, beta-oxidation, adipogenesis, and adipocyte differentiation [7].

Although NAFLD is showing increasing prevalence worldwide, there is no approved effective drug therapy and the current disease management plan depends primarily on the reduction of body weight, exercise, and lifestyle modification [11, 12]. However, in view of the prominent role of PPARs in the regulation of lipid metabolism and glucose homeostasis, it is not unexpected that this group of nuclear receptors is the focus of the drug development research of NAFLD treatment [13, 14].

Experimental research and preliminary clinical trials suggest a protective role of PPAR agonists in NAFLD and NASH through multiple mechanisms of action including stimulating the expression of the genes of fatty acid beta-oxidation and suppressing the genes of inflammation and oxidative stress [14, 15]. As a point of fact, therapeutic utilization of the pharmacological agonists of PPAR-*α* and PPAR-*γ* showed promising results in reducing IR and inflammation and interrupting the pathogenesis of DM and NAFLD in animal models and human patients [14–17]. However, there are considerable adverse effects and the ideal agonist is not yet available [18, 19].

Camel milk (CM) has a unique composition rich in immunoglobulins, vitamins, and trace elements such as magnesium, zinc, manganese, and selenium etc. In addition, it fosters the absorption and metabolism of vitamins B, C, and E that have protective effects against oxidative stress damage of the cells [20]. Moreover, CM has high levels of insulin, insulin-like proteins, and L-carnitine, and it stimulates the release of incretin hormones in diabetic animals [21, 22]. The peculiar composition of CM was associated with beneficial effects in NAFLD including decreased appetite, diminished cholesterol absorption from the gut, and reduced fat accumulation in the liver. Additionally, CM treatment counteracted hyperglycemia, IR, oxidative stress, and inflammation in experimental models of DM [22–25].

Many of the reported effects of CM treatment in patients and animal models of DM and NAFLD including the hepatoprotective, antihyperlipidemic, insulin-sensitizing, antioxidative, and anti-inflammatory actions [21, 24, 25] cross-match with the stated actions of PPAR ligands and agonists in the treatment of these diseases [13–17, 26, 27].

Therefore, the current study hypothesized that CM may produce some or all of its beneficial effects in NAFLD through modifying the expression and/or the actions of PPARs regulating the fat metabolism and energy balance. However, up to the best of our knowledge, the effects of CM treatment on PPARs have not yet been studied in either the normal or pathological states. This stimulated our interest to examine the effects of CM treatment on the expression of PPARs (*α* and *γ*), carnitine palmitoyl-transferase-1 (CPT1A), and fatty acid-binding protein-1 (FABP1) in the liver, heart, and kidney tissues in a rat model of NAFLD induced by high-fat cholesterol-rich diet (HFD-C) intake. Additionally, the changes in the serum levels of the inflammatory cytokines interleukin-6 (IL-6) and tumor necrosis factor-alpha (TNF-*α*) and the adipokines leptin and adiponectin were also investigated.

## 2. Materials and Methods

### 2.1. Animals and Experiment Protocol

The study involved forty male Wistar rats, 6 to 8 weeks old (weighing 270–325 g), obtained from the Experimental Animal Care Unit of the College of Medicine, King Khalid University Hospital, King Saud University (KSU). Animals were housed 4 per cage under standard laboratory conditions of a controlled temperature of 21-23°C and 60% humidity in a 12 h light/dark cycle with free access to standard rodent chow and sterile drinking water. The study protocol was revised and accepted by the institutional review board (IRB) of KSU. The experimental techniques followed the international guidelines of the use and care of the laboratory animals and the regulations of the Experimental Animal Care Unit of the College of Medicine, KSU. The animals were randomly divided into four experimental groups (*n* = 10 in each): control group: control healthy animals receiving no camel milk treatment; control+CM group: control healthy animals receiving camel milk treatment; NAFLD group: animals with nonalcoholic fatty liver disease (NAFLD) receiving no treatment; and NAFLD+CM group: animals with NAFLD treated with camel milk.

### 2.2. Induction of NAFLD by a High-Fat Cholesterol-Rich Diet (HFD-C)

The animals in the control and control+CM groups received a commercial ordinary chow diet composed of carbohydrates (55%), proteins (20%), fats (4%), fibers (3.5%), and ash (6%); iron, calcium, phosphorous, vitamins A, D, and E, and trace elements cobalt, copper, iodine, manganese, selenium, and zinc were purchased from Grain Silos & Flour Mills Organization, Riyadh Branch, Riyadh, Saudi Arabia. The animals in the NAFLD and NAFLD+CM groups received a high-fat cholesterol-rich diet (HFD-C), in which 42% of the energy is derived from fats by the addition of 1.5% cholesterol (Sigma-Aldrich, USA) and 8% coconut oil to the basal diet [25].

### 2.3. Collection and Administration of Camel Milk

Camel milk was collected from the *Camillus dromedaries* breed in a private camel farm located outside Riyadh city, Saudi Arabia. In an attempt to keep the composition and quality of the used milk, the food type and the time of milking of the camels were fixed throughout the study. The camels were milked daily in the early morning by the traditional milking technique under sanitary conditions in sterile screw-capped containers. The collected milk was kept immediately in refrigerated boxes and transferred to the laboratory. We conducted a pilot study to determine the amount of milk that could be taken by the experimental animals per day. Accordingly, the animals in the control+CM and NAFLD+CM groups received oral camel milk (50 ml/kg/day) for 8 weeks.

### 2.4. Blood and Tissue Sampling

At the end of the study, the animals were weighed and deprived of food but allowed to drink water the night before the samples collection. At the time of sampling, the animals received Nembutal anesthesia (50 mg/kg by intraperitoneal injection) [22].

The blood was collected into plain test tubes by cardiac puncture; then, the animals were sacrificed by decapitation, and the liver, heart, and kidney tissues were isolated, washed with cold saline, sliced into small pieces, placed into liquid nitrogen, and transferred to a -80°C freezer to be stored for western blot studies. The serum was separated and stored at -20°C for further biochemical analysis.

### 2.5. Western Blot Studies

Protein extracts were prepared from the thawed liver, heart, and kidney tissue samples. Equal amounts of proteins were separated on 10% sodium dodecyl sulfate-polyacrylamide gel electrophoresis (SDS-PAGE) (TGX™ FastCast™ Acrylamide Kit, 12% NO. 1610175). The tissues were then transferred onto polyvinylidene difluoride (PVDF) membranes (Trans-Blot® Turbo™ Mini PVDF Transfer Packs NO. 1704156; Bio-Rad, USA) and were subsequently blocked with nonfat dry milk (Blotting-Grade Blocker NO. 1706404 for western blot applications). The membranes were incubated with primary antibodies against PPAR-*α* (ab24509; Abcam, USA), PPAR-*γ* (ab209350; Abcam, USA), CPT1A (ab83862; Abcam, USA), liver FABP antibody-N-terminal (ab190958; Abcam, USA), and glyceraldehyde 3-phosphate dehydrogenase (GAPDH) (ab181602; Abcam, USA). After washing with 0.1% Tween 20 in Tris-buffered saline (TBS), the membranes were incubated with the horseradish peroxidase-conjugated secondary antibody (ab6721; Abcam, USA). The images were detected by the ChemiDoc MP System imager. The bands were quantified and analyzed by JLab software.

### 2.6. Enzyme-Linked Immunosorbent Assay (ELISA) of Cytokines and Adipokines

The serum levels of IL-6, TNF-*α*, leptin, and adiponectin were determined by the sandwich enzyme immunoassay technique. The commercial rat ELISA kits for IL-6 (SEA079Ra), TNF-*α* (SCA133Ra), leptin (SEA084Ra), and adiponectin (SEA605Ra) were purchased from Cloud-Clone Corporation Inc. (Katy, TX, 77494, USA). The technique of the assay was according to the manufacturer's instructions.

### 2.7. Statistical Analysis

The data was tested for normal distribution and statistically analyzed by GraphPad Prism 9.0 software. Multiple group comparison for each studied parameter was carried out by one-way analysis of variance (ANOVA), and Tukey's post hoc test identified the statistically significant groups. Results were considered significant at *p* < 0.05.

## 3. Results

### 3.1. PPAR-*α*

**Figure 1:**
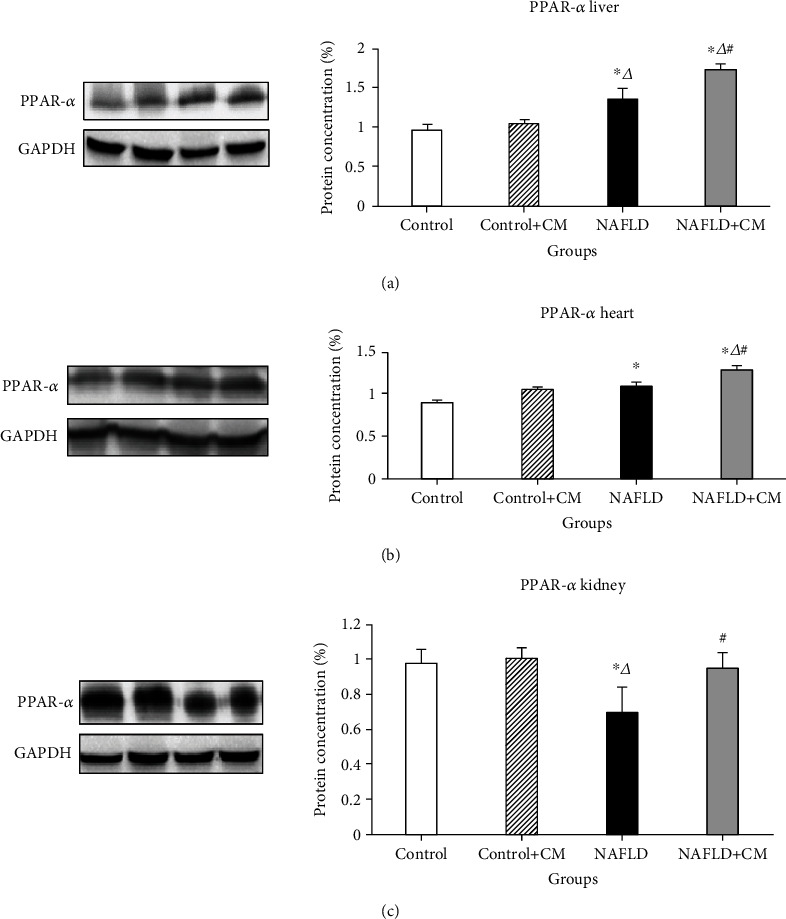
PPAR-*α* protein expression in the liver (a), heart (b), and kidney (c) tissues of the control, camel milk (CM) treated control (control+CM), nonalcoholic fatty liver disease (NAFLD), and CM-treated NAFLD (NAFLD+CM) animals. The protein bands were quantified relative to GAPDH. ^∗^*p* < 0.05 versus the control group, ^∆^*p* < 0.05 versus the control+CM group, and ^#^*p* < 0.05 versus the NAFLD group.

Western blot studies showed increased liver PPAR-*α* protein concentration in the NAFLD group in comparison to the control group (*p* = 0.0015) (Figure 1(a)). At the same time, CM treatment for 8 weeks exerted further upregulation of the PPAR-*α* in the liver of the NAFLD+CM group in comparison to the NAFLD group (*p* = 0.0016). There was no significant change of the PPAR-*α* proteins in the liver of the healthy control+CM group receiving CM in comparison to the non treated control group (*p* > 0.05). High-fat diet intake was also associated with increased (*p* = 0.028) PPAR-*α* in the heart of the NAFLD group in comparison to the control group receiving normal chow diet (Figure 1(b)). The expression of PPAR-*α* was higher (*p* = 0.017) in the heart tissue of the animals in the NAFLD+CM group in comparison to the NAFLD group. Alternatively, the kidney showed a significant decrease (*p* = 0.028) in the PPAR-*α* protein levels in the NAFLD group in comparison to the control group (Figure 1(c)). However, the kidney PPAR-*α* protein was higher (*p* = 0.014) after CM treatment in the NAFLD+CM group in comparison to the NAFLD group.

### 3.2. PPAR-*γ*

**Figure 2:**
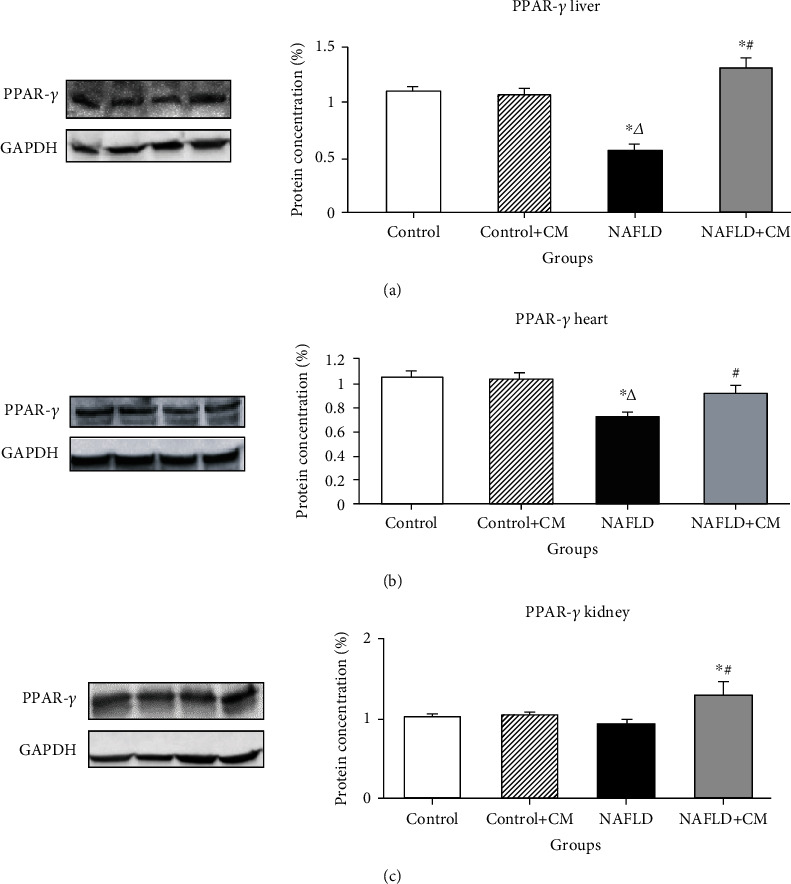
PPAR-*γ* protein expression in the liver (a), heart (b), and kidney (c) tissues of the control, camel milk (CM) treated control (control+CM), nonalcoholic fatty liver disease (NAFLD), and CM-treated NAFLD (NAFLD+CM) animals. The protein bands were quantified relative to GAPDH. ^∗^*p* < 0.05 versus the control group, ^∆^*p* < 0.05 versus the control+CM group, and ^#^*p* < 0.05 versus the NAFLD group.

The proteins of PPAR-*γ* showed decreased expression in the liver of the NAFLD group in comparison to the control group (*p* = 0.001) (Figure 2(a)). The effect of NAFLD on the hepatic PPAR-*γ* was reversed by camel milk treatment in the NAFLD+CM group which showed greater levels (*p* < 0.0001) of PPAR-*γ* proteins in comparison to the NAFLD group. The NAFLD was also associated with decreased (*p* = 0.001) PPAR-*γ* in the heart of the NAFLD group in comparison to the control group (Figure 2(b)). However, CM treatment effectively stimulated (*p* = 0.0123) the PPAR-*γ* proteins in the cardiac tissue of the NAFLD+CM group in comparison to the NAFLD group. There was a slight nonsignificant (*p* > 0.05) decrease of PPAR-*γ* in the kidney tissues of the NAFLD group in comparison to the control group (Figure 2(c)). Nevertheless, CM treatment successfully stimulated (*p* = 0.0059) the expression of the renal PPAR-*γ* in the NAFLD+CM group in comparison to the NAFLD group.

### 3.3. CPT1A

**Figure 3:**
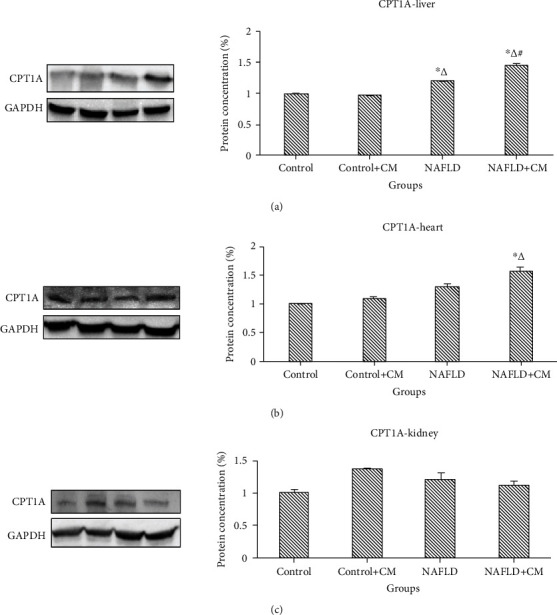
CPT1A protein expression in the liver (a), heart (b), and kidney (c) tissues of the control, camel milk (CM) treated control (control+CM), nonalcoholic fatty liver disease (NAFLD), and CM-treated NAFLD (NAFLD+CM) animals. The protein bands were quantified relative to GAPDH. ^∗^*p* < 0.05 versus the control group, ^∆^*p* < 0.05 versus the control+CM group, and ^#^*p* < 0.05 versus the NAFLD group.

Similar to the PPAR-*α*, the CPT1A proteins increased (*p* < 0.0001) in the hepatic tissues of the NAFLD group in comparison to the control group (Figure 3(a)). Camel milk treatment induced further upregulation (*p* < 0.0001) of CPT1A levels in the hepatic tissues of the NAFLD+CM group in comparison to the nontreated NAFLD group. However, there was no significant change in the CPT1A expression in the cardiac or renal tissues in the NAFLD group (*p* > 0.05). Camel milk treatment increased CPT1A levels in the heart of the NAFLD+CM group compared to the control group (*p* = 0.007) (Figure 3(b).

### 3.4. FABP1

**Figure 4:**
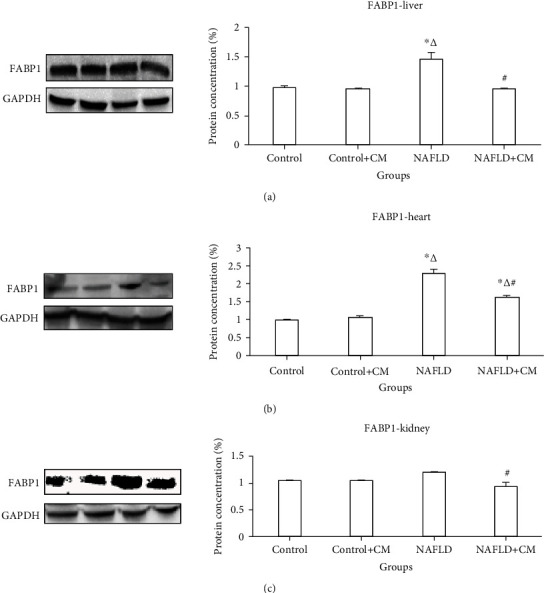
FABP1 protein expression in the liver (a), heart (b), and kidney (c) tissues of the control, camel milk (CM) treated control (control+CM), nonalcoholic fatty liver disease (NAFLD), and CM-treated NAFLD (NAFLD+CM) animals. The protein bands were quantified relative to GAPDH. ^∗^*p* < 0.05 versus the control group, ^∆^*p* < 0.05 versus the control+CM group, and ^#^*p* < 0.05 versus the NAFLD group.

The NAFLD group showed increased (*p* < 0.0001) FABP1 in the liver and heart tissues in comparison to the control group (Figures 4(a) and 4(b)). The renal FABP1 level showed no significant change in the NAFLD group in comparison to the control group (*p* > 0.05) (Figure 4(c)). However, CM treatment decreased the FABP1 proteins in the hepatic, cardiac, and renal tissues (*p* < 0.0001, *p* = 0.0003, and *p* = 0.007, respectively) of the NAFLD+CM group in comparison to the NAFLD group.

### 3.5. The Inflammatory Cytokines

**Figure 5:**
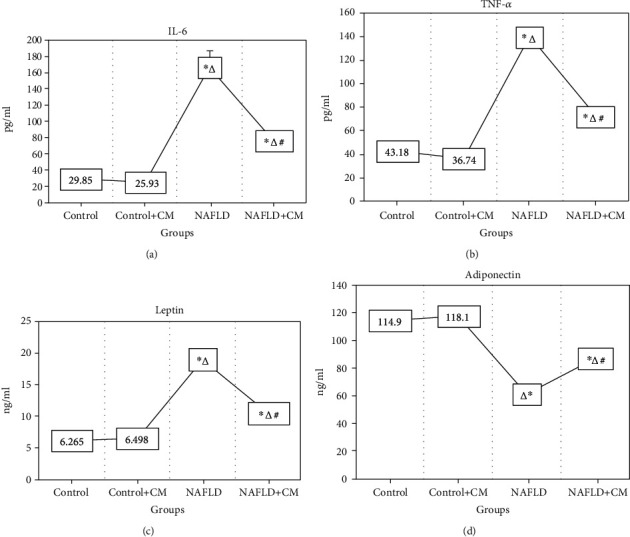
The serum levels of IL-6 (a), TNF-*α* (b), leptin (c), and adiponectin (d) in the control, camel milk (CM) treated control (control+CM), nonalcoholic fatty liver disease (NAFLD), and CM-treated NAFLD (NAFLD+CM) animals. ^∗^*p* < 0.05 versus the control group, ^∆^*p* < 0.05 versus the control+CM group, and ^#^*p* < 0.05 versus the NAFLD group.

The prolonged ingestion of HFD-C leads to a proinflammatory-like condition in the NAFLD group manifested by increased (*p* < 0.0001) serum IL-6 and TNF-*α* levels in comparison to the control group (Figures 5(a) and 5(b)). Camel milk treatment abolished the inflammatory response induced by HFD-C and diminished (*p* < 0.0001) the serum levels of the inflammatory cytokines in the NAFLD+CM group in comparison to the NAFLD group.

### 3.6. Serum Leptin and Adiponectin

In association with the increased inflammatory cytokines TNF-*α* and IL-6, the NAFLD group showed significant increases in the serum leptin levels (*p* < 0.001) and decreased adiponectin production (*p* < 0.0001) in comparison to the control group (Figures 5(c) and 5(d)). The amelioration of hyperlipidemia and decreased body weight after CM treatment (data not presented) were accompanied by a significant decrease (*p* < 0.0001) in serum leptin and increased (*p* < 0.0001) circulating adiponectin levels in the NAFLD+CM group in comparison to the NAFLD group.

## 4. Discussion

PPARs are key modulators in the pathological course of NAFLD and are also candidate targets for treating the disease [7].

### 4.1. The Effects of CM Treatment

The beneficial effects of CM treatment were reported in numerous acute and chronic health problems including acute paracetamol hepatotoxicity, carbon tetrachloride-induced liver failure, NAFLD, food-induced allergy, DM, bronchial asthma, atherosclerosis, and autism [22, 23]. Using the size exclusion chromatography (SEC), our research group recently separated small peptide fractions (SEC-1 and SEC-2) of the papain-hydrolyzed camel whey protein. These peptides exerted significant antioxidant activities and inhibition of the angiotensin-converting enzyme. The smaller size fraction (SEC-1) exerted powerful hepatoprotective, antihyperlipidemic, and antioxidant effects in thioacetamide-induced hepatotoxicity [24]. In a recent publication of our research group, we reported the protective effects of CM treatment on the histological and biochemical features of NAFLD induced by HFD-C in rats [25]. Camel milk decreased the steatosis, ballooning degeneration, and inflammatory cellular infiltration of hepatocytes. Furthermore, the CM-treated animals showed improved lipid profile, decreased IR, and enhanced glucose tolerance. Additionally, the antioxidant properties of CM increased the catalase activity and decreased the lipid peroxidation product malondialdehyde formation in the treated animals [25]. Many of the effects of CM treatment in NAFLD matched with the actions of the PPAR-*α* agonist (fibrates), the thiazolidinediones (TZDs) stimulating PPAR-*γ*, the dual *α*/*γ* agonist (glitazars), and the latest PPAR-*α*/*δ* agonist (elafibranor) in NAFLD and in obese patients with IR and DM as part of the metabolic syndrome. The latter drugs decrease IR, glucose intolerance, and inflammatory response [14–16, 27, 28].

In view of the aforementioned evidences, we hypothesized that CM produces its protective effects in the HFD-C-induced NAFLD through modifying the PPAR expression and/or actions in the metabolically active tissues associated with the energy balance and fat metabolism.

### 4.2. The Changes of PPAR-*α*/*γ*, CPT1A, and FABP1 in NAFLD

The findings of the present study revealed increased protein levels of PPAR-*α*, CPT1A, and FABP1 and decreased PPAR-*γ* in the hepatic, cardiac, and renal tissues of the NAFLD animals. These results coincide with similar reports of increased expression of PPAR-*α* and its downstream genes mediating fat metabolism in wild-type mice receiving HFD [29, 30]. The increased PPAR-*α* and the downstream primary regulator enzyme and transporter protein CPT1A in the NAFLD animals could be an adaptive response to the excessive lipid input resulting from the HFD-C administration to enhance the FFA entry into the cells for beta-oxidation [31, 32].

### 4.3. The effect of CM Treatment on PPAR-*α* and CPT1A

Western blot studies showed that the administration of CM treatment to the NAFLD+CM group of animals boosted the hepatic and extrahepatic expression of PPAR-*α*, PPAR-*γ*, and CPT1A and normalized the FABP1 proteins. The increased expression of PPARs (*α*, *γ*) and CPT1A is anticipated to enhance FA metabolism, inhibit lipolysis, and stimulate adipogenesis in the treated animals [32]. The extrahepatic PPAR-*α* plays a role in the general body fat homeostasis, while the presence of normal hepatic PPAR-*α* is essential for the prevention of liver steatosis, as evidenced by the development of NAFLD in nonobese mice lacking hepatocyte PPAR-*α* with aging [33]. The enhanced PPAR-*α* expression protects against steatosis and steatohepatitis by facilitating the hepatic uptake of the circulating lipids, stimulating the peroxisomal and mitochondrial FA oxidation, and suppressing a number of the inflammatory genes [16, 29, 34]. It is also reported that PPAR-*α* induces the expression of the liver-derived hormone fibroblast growth factor 21 (FGF21) which has hepatoprotective and multiple endocrine actions [35].

The current findings of the hepatoprotective effects of CM associated with the increased PPAR-*α* and CPT1A protein levels coincide with similar results in the NAFLD mice treated with the natural sweetener stevioside extracted from the medicinal plant *S*. *rebaudiana* Bertoni. The stevioside-treated animals showed hypolipidemic and antisteatotic effects that were attributed to the stimulation of PPAR-*α* and CPT1A expression and actions in the liver [36].

Likewise, the drugs that activate PPAR-*α* through hydroxymethylation such as the ten-eleven translocation-1 (TET1) enzyme exerted protective effects against NAFLD, stimulated FA oxidation, and suppressed triglyceride accumulation in the liver [37]. Furthermore, patients with NAFLD showed increased expression of PPAR-*α* in direct association with the histological improvement of the disease after lifestyle modification or surgical interventions of obesity [38].

### 4.4. FABP1 in NAFLD and the Effect of CM Treatment

In healthy liver conditions, FABP1, also known as liver FABP (LFABP), is involved with PPARs in the intracellular FA transport and cholesterol and phospholipid metabolism, and has scavenging actions that protect the cells from oxidative damage [39]. However, due to its small molecular weight (15 kDa) and its intracellular location, FABP1 is released in the serum in increased quantities in several pathological conditions involving hepatocyte injury and was reported to have a pathogenic role in NAFLD in diabetic patients [40]. Additionally, FABP1 was considered an early biomarker that determines the extent of fatty liver infiltration in NAFLD patients through increasing steatosis and subsequent activation of the hepatic stellate cells [41].

The increased expression of FABP1 in the liver and heart of the NAFLD group of animals in the present study indicates that the pathology of NAFLD involves the hepatic and cardiac tissues. The administration of CM treatment ameliorated the histological and biochemical picture of NAFLD, as we reported previously [25]. Furthermore, it normalized the FABP1 levels in the NAFLD+CM group of animals. This supports previous reports of the direct relationship of the FABP1 levels and the severity of NAFLD [41].

### 4.5. The Hypolipidemic and Antisteatotic Effects of CM

We recently reported decreased serum cholesterol, triglyceride, LDL-C, VLDL-C, and hepatic fat accumulation in NAFLD animals receiving CM treatment [25]. To gain insight into the concrete mechanisms of the hypolipidemic and antisteatotic effect of CM, we examined the hepatic and extrahepatic expression of PPAR-*γ* in the studied groups. PPAR-*γ* is known as an “energy balance receptor” and is a crucial regulator of many PPRE-containing genes such as FABP4 and CPT1A having an essential function in fat metabolism and lipogenesis, resulting in the decline of the circulating blood lipids and inhibition of the liver steatosis [6, 14–17].

In this study, the expression of PPAR-*γ* decreased in liver and cardiac tissues of the NAFLD group compared to the control group. Meanwhile, the NAFLD+CM group showed recovery of the normal PPAR-*γ* protein levels in the hepatic and extrahepatic tissues leading to decreased steatosis and improved blood lipid profile.

The present results are in accordance with recent studies that reported decreased IR, hepatic steatosis, and inflammatory reactions in animals receiving HFD after the restoration of the normal PPAR-*γ* expression by swimming exercises and palmitoleic acid supplementation [42, 43].

### 4.6. Insulin Resistance

The insulin-sensitizing agents are carrying promising prospects for IR that characterize the patients of NAFLD [16]. Interestingly, our reported findings showed improved glucose tolerance marked by decreased fasting and postprandial glucose levels and HOMA-IR in NAFLD animals treated with CM in comparison to the nontreated NAFLD group [25]. This could be explained by the increased tissue expression of PPAR-*γ* in the hepatic and cardiac tissues of the NAFLD+CM animals in the present study. It was reported that PPAR-*γ* mediated increased insulin action and sensitivity in animals and humans with increased IR [16, 42]. The stimulated PPAR-*γ* activity induces the signaling molecules such as c-CBL-associated proteins of insulin receptor substrate-2 (IRS2) and downregulates the local glucocorticoid actions. This leads to increased hepatic response to insulin-mediated inhibition of glucose production and stimulation of muscle glucose uptake, storage, and metabolism [16, 28, 42].

In their in vitro studies on the antimitogenic and anticancer effects of PPAR-*γ* and its agonists, Costa et al. reported that TZDs stimulated the PPAR-*γ* expression and suppressed cancer cell proliferation. They related the anticancer actions of TZDs to a pleiotropic effect of PPAR-*γ* that inhibits the insulin receptor gene in the HepG2 cells that have an abnormally high density of insulin receptors [44]. In the same subject, Corigliano et al. associated the anticancer effect of PPAR-*γ* agonists to the inhibition of cell adhesion, stimulation of apoptosis, and suppression of inflammation by increased adiponectin [45]. This supports the use of TZDs as adjuvant anticancer therapy. Similarly, in the current study, camel milk stimulated the expression of PPAR-*γ* and PPAR-*α* in the HFD-C-induced NAFLD and exerted hypolipidemic and anti-inflammatory effects demonstrated by increased adiponectin and decreased IL-6, TNF-*α*, and leptin levels. This was associated with decreased hepatic steatosis and degeneration of the hepatocytes, increased glucose tolerance, and inhibition of IR. It is worth noting that camel milk was recently reported to exert anticancer effects against several types of cancer cells including colorectal and breast cancer through stimulating autophagy [46], apoptosis [47], antioxidant effects [48], and inhibition of the proinflammatory, proangiogenic, and profibrogenic cytokines [49], modifying the expression of cancer-activating and cancer-protective genes [47]. The present findings of activation of PPARs (*α* and *γ*) by camel milk may add another mechanism of the anticancer effects of camel milk treatment, but this needs further in vitro and in vivo studies.

### 4.7. Cytokines and Adipokines in NAFLD

The animals with NAFLD in the present study showed a proinflammatory-like condition characterized by increased IL-6, TNF-*α*, and leptin levels together with decreased production of the anti-inflammatory adiponectin. This was in accordance with the reported changes of these cytokines in obesity, IR, type II DM, and atherosclerosis which are components of the metabolic syndrome [50]. The increased inflammatory markers IL-6 and TNF-*α* foster the transition from simple NAFLD to NASH [5], and the high serum leptin level reflects leptin resistance and predicts the degree of fibrosis in NAFLD [51]. However, the efficient anti-inflammatory effect of adiponectin works to dampen the obesity-linked inflammatory changes in the liver [52].

### 4.8. The Anti-Inflammatory Effect of CM Treatment

The anti-inflammatory properties of camel milk inhibited the inflammatory cytokines and leptin production and increased adiponectin in the NAFLD+CM group of animals. This could be ascribed to the activation of PPAR-*α* which downregulates the genes of nuclear factor kappa B (NF-*κ*B), TNF-*α*, and toll-like receptor (TLR) signaling pathways related to inflammation [33]. Additionally, the CM-induced activation of adiponectin release is suggested to stimulate adiponectin receptor 2 (adipoR2) leading to activation of adenosine monophosphate-activated protein kinase (AMPK) signaling and PPAR-*α* that culminates with the CM-induced stimulation of PPAR-*γ* activity in the suppression of a plethora of inflammatory cytokines including IL-6, TNF-*α*, and IL-1 ending up with damping the inflammation [53, 54].

The anti-inflammatory effect of CM in animals with NAFLD coincides with the action of the PPAR-*α* agonists fibrates [55] and the PPAR-*γ* agonists, e.g., TZDs and troglitazone, that inhibit TNF-*α* expression and action in adipocytes and inhibit TNF-*α*-mediated IR [56].

## 5. Conclusion

We conclude that camel milk treatment stimulates the expression of PPARs (*α*, *γ*) and CPT1A and increases adiponectin release. At the same time, it suppresses FABP1, TNF-*α*, IL-6, and leptin levels in NAFLD+CM animals. These mechanisms enhanced lipid uptake and metabolism in the hepatic and extrahepatic tissues and improved glucose tolerance in the NAFLD+CM animals. The CM-mediated activation of PPARs (*α*, *γ*) hindered steatohepatitis, hyperlipidemia, and IR. The current results strongly support the beneficial effects of camel milk in counteracting the deleterious effects of HFD-C on lipid metabolism and glucose homeostasis. Camel milk treatment constrained NAFLD and can protect against the components of the metabolic syndrome including hepatic steatosis, IR, and DM in high-risk obese patients. Nevertheless, through acting as an agonist to PPARs (*α* and *γ*), camel milk and its bioactive molecules can provide a safe natural alternative of currently unknown side effects to the pharmacological PPAR ligands (such as fibrates and TZDs). This can help to alleviate the risk of the adverse effects of the long-term use of these drugs in diabetic and obese patients requiring prolonged durations of therapy.

## Data Availability

The data is available upon request.
